# Effects of emotional intelligence on physical activity engagement and the mediating roles of achievement motivation and interpersonal relationship in Chinese undergraduate students

**DOI:** 10.3389/fpubh.2024.1476150

**Published:** 2024-11-13

**Authors:** Chengfeng Yu, Aochuan Xue, Zhaohong Zeng, Qianjin Wu

**Affiliations:** ^1^School of Physical Education, Shandong University, Jinan, Shandong, China; ^2^School of Physical Education and Health, East China Normal University, Shanghai, China; ^3^School of Sports and Health, Zunyi Medical University, Zunyi, Guizhou, China

**Keywords:** emotional intelligence, physical activity engagement, achievement motivation, interpersonal relationships, structural equation modeling

## Abstract

**Background:**

Engaging in physical activity has been demonstrated to enhance cardiorespiratory fitness, muscle strength, bone health, and cardiometabolic health. However, it is concerning that more than 80% of students globally do not meet the recommended standard of at least 1 h of physical activity per day. It is imperative to enhance student involvement in physical activities.

**Objective:**

This study aims to investigate the effect of emotional intelligence (EI) on undergraduate students’ physical activity engagement (PAE), to elucidate the mediating role of achievement motivation (AM) and interpersonal relationships (IR).

**Methods:**

A stratified whole-sample was used to survey 810 university students (19.84 ± 1.40 years). The scale items were designed to assess the participants’ EI, PAE, AM, and IR based on the correlation scale. SPSS and AMOS were used to analyze the mediating effect.

**Results and conclusion:**

The results demonstrated a positive effect of EI on AM and PAE (*β* = 0.29, *p* < 0.001; *β* = 0.28, *p* < 0.001). Furthermore, AM was found to have a positive effect on PAE (*β* = 0.07, *p* < 0.05). Notably, AM mediated between EI and PAE. In conclusion, our findings provide further insight into the effect of EI on undergraduate students’ PAE, and reveal the mediating role of AM.

## 1 Introduction

Physical activity can be defined as any bodily movement undertaken with the intention of promoting health and well-being (1). It is of the utmost importance to engage in physical activity in order to maintain optimal health and well-being. It has been demonstrated to extend longevity and enhance the quality of life (2, 3). However, the World Health Organization has indicated that one in four adults and four in five adolescents are not engaging in sufficient physical activity and that more than 80% of students globally are not meeting the current recommendation of at least 1 h of physical activity per day (4, 5). Physical inactivity has become the fourth leading risk factor for death worldwide, after hypertension, smoking, and hyperglycemia. Individuals who are physically inactive have a 20 to 30 percent increased risk of death (6, 7). Furthermore, it is the most significant public health problem of the twenty-first century. A deficiency in physical activity can be defined as a lack of sufficient physical activity engagement (PAE). PAE is used to describe the psychological identification of individuals with physical activity. This identification is based on the individual’s physiological involvement, cognitive arousal, and emotional state of sensitivity when in PAE (8). It is therefore recommended that the antecedent mechanisms of PAE and the factors that increase interest and willingness to engage in physical activity be explored, as this will help to obtain more benefits from exercise and is an effective solution to address physical inactivity.

In recent years, changes in living habits and increased study pressure have resulted in a significant proportion of university students adopting sedentary lifestyles characterized by limited movement and a lack of outdoor exercise (4, 9). Furthermore, the level of PAE among this demographic is generally deemed to be unsatisfactory. Previous research has demonstrated that over 80% of adolescents enrolled in schools worldwide fail to meet the current recommended threshold of at least 1 h of physical activity per day, and more specifically, 85% of girls and 78% of boys do not meet this standard (5, 10). The promotion of physical and mental health through increased PAE is a topic that has become a common point of concern for various parties, including parents, educational institutions, the general public, and researchers.

In light of the interdisciplinary developments that have occurred over time, researchers have endeavored to elucidate the attribution of PAE and improvement strategies from a multitude of perspectives (11). Among these, the role of psychology has gradually come to occupy a more prominent position, with evidence mounting that it constitutes an important factor influencing PAE (11, 12). For instance, emotional intelligence (EI) is a significant element in the evaluation of athletes’ psychological well-being. It is predominantly employed to investigate its influence on athletic performance (13) and motivation (14). Additionally, it is utilized to assess the mental states of athletes across diverse sporting disciplines (15, 16). EI is the capacity to perceive, understand, and manage emotions in oneself and others. It encompasses the ability to discern and utilize emotional information to inform one’s thoughts and actions, thereby regulating one’s own emotions and facilitating the successful completion of tasks. Those with elevated levels of EI are more likely to PAE due to their capacity to regulate emotions, establish objectives, and cultivate positive interpersonal connections (17, 18).

Achievement motivation (AM) is the psychological process that inspires, motivates, guides, and maintains behavior and performance and refers to the driving force that an individual generates when faced with a task, a goal, or a challenge, and it is a state of mind that strives for success, surpasses difficulties or achieves excellent performance (19–21). The process of self-regulation in PAE, whereby EI and AM work in conjunction to enable individuals to modify their psychology and behavior in order to achieve personal, collective, and organizational objectives, constitutes a significant psychological factor that may exert an influence on the participation of university students in PAE (22, 23). Thus, AM may be an important ‘bridge’ for EI to influence university students’ PAE.

The formation of interpersonal relationships (IR) represents the foundation of human social interaction. The establishment of IR also serves to fulfill the individual’s fundamental needs for a sense of belonging, intimacy, and communication with others (24, 25). EI enables us to gain a deeper understanding of our own experiences and to develop the wisdom to comprehend the behavior of others, discern their emotions, and identify their intentions (26, 27). This facilitates our ability to cope with unexpected situations and difficulties in our personal and professional relationships (28, 29). EI is a desirable quality that shapes an individual’s IR and is conducive to the formation and maintenance of IR.

In conclusion, despite the existence of significant health issues related to physical inactivity among university students for decades, conventional strategies for increasing physical activity participation have yielded limited results. It is therefore necessary to consider the psychological factors that influence PAE and the effectiveness of improvement strategies. The aim of this study is to make a more contribution to the field of PAE. By investigating the relationship between EI and PAE, we aim to gain insight into the underlying mechanisms that drive this relationship and provide a scientific foundation for the practice of improving PAE.

## 2 Aims and hypothesis

The research was designed to achieve the following aims:

(1) Investigating the effects of EI on university students’ PAE.
(2) Uncovering the Mediating Role of AM and IR between EI and PAE.

Based on these aims, seven hypotheses were established:

*H1*: EI significantly and positively affects university students’ PAE.

*H2*: EI significantly and positively affects university students’ AM.

*H3*: AM significantly and positively affects university students’ PAE.

*H4*: AM mediates the influence of EI on university students’ PAE.

*H5*: EI significantly and positively affects university students’ IR.

*H6*: IR significantly and positively affects university students’ PAE.

*H7*: IR mediates the influence of EI on university students’ PAE.

**Figure 1:**
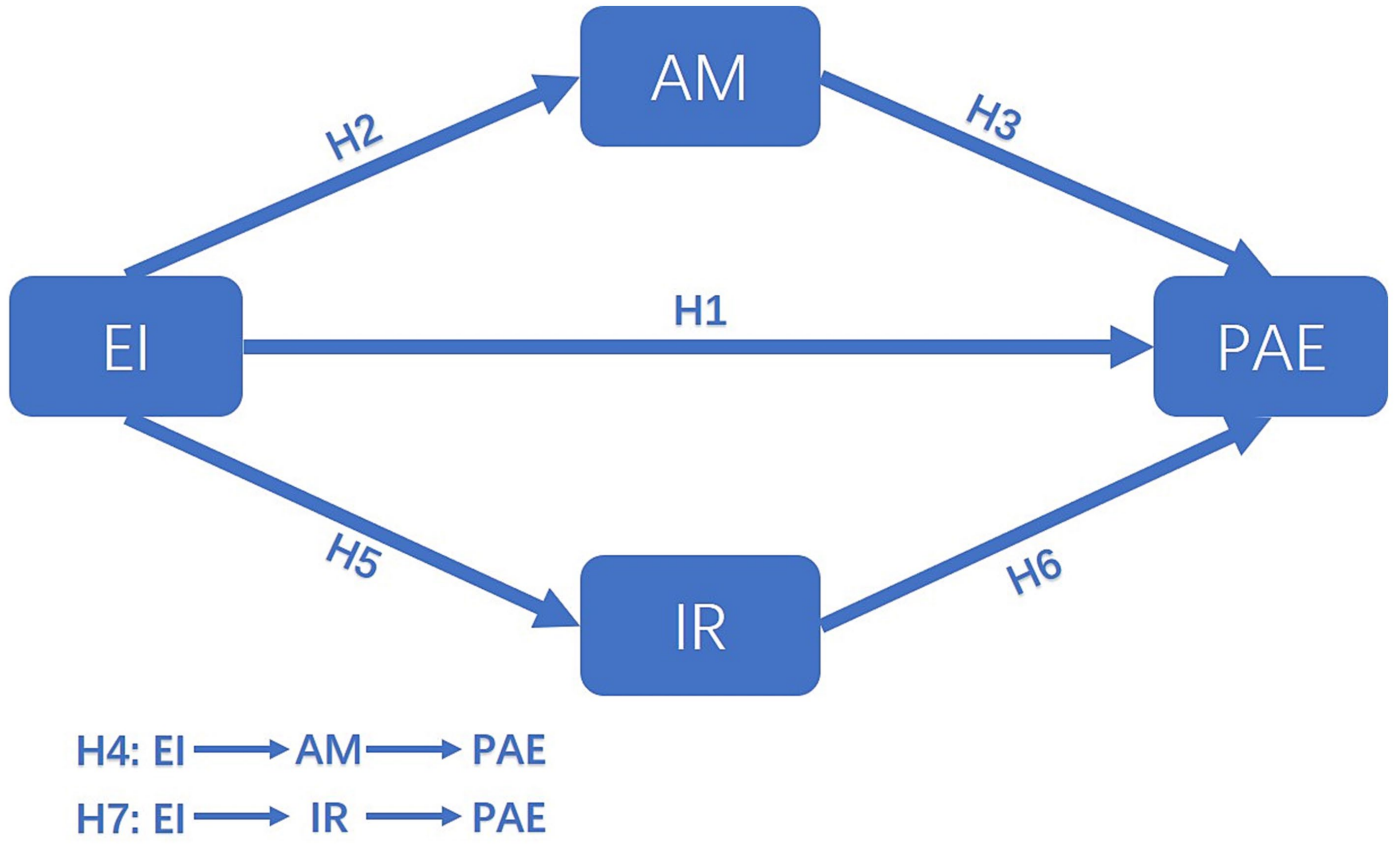
Conceptual model. EI, emotional intelligence; AM, achievement motivation; IR, interpersonal relationships; PAE, physical activity engagement.

The mediation model, which synthesizes the research hypotheses, is shown in Figure 1.

## 3 Materials and methods

### 3.1 Study design

This study employed a cross-sectional research design to examine the influence of EI on physical activity engagement and the mediating function of AM and IR among Chinese undergraduate students. The research was conducted in accordance with the ethical standards set forth in the Declaration of Helsinki and received approval from the Ethics Committee of Shandong University. All participants provided written informed consent.

### 3.2 Participants

This study used the technique of stratified overall sampling to conduct a questionnaire survey in June 2024 at four universities in Shandong Province, targeting undergraduate students in their first to third years of study. The questionnaires were distributed via WeChat, Tencent QQ, and other social media platforms. A total of 1,206 questionnaires were recovered, and following the removal of invalid questionnaires and the application of data cleaning procedures, 810 valid questionnaires were obtained (19.84 ± 1.40 years), representing a questionnaire validity rate of 67.2%.

The mean age of the participants was 19.84 ± 1.40 years. Participants questionnaires will be excluded in the following cases: (1) Incomplete questionnaires, defined as those in which a significant number of sections are not completed. (2) Questionnaires in which the respondents demonstrated a lack of comprehension of the questionnaire’s content and provided erroneous responses, or questionnaires in which the respondents did not adhere to the instructions, such as skipping questions that did not align with the desired format. (3) Questionnaires exhibiting minimal variability in responses, such as those employing a 5-point attitude scale where the respondent selects the midpoint (3) irrespective of whether their perceptions are positive or negative. (4) Questionnaires that are incomplete or illegible. (5) Questionnaires that are returned after the stipulated deadline. (6) Questionnaires completed by individuals who do not meet the requisite criteria, such as those completed by individuals who are not university students, should be considered invalid. (7) Questionnaires that are inconsistent or contain obvious errors.

### 3.3 Measures

#### 3.3.1 Emotional intelligence scale

In this study, we employ the Emotional Intelligence Scale developed by Schutte (30, 31), as a measure of emotional intelligence among university students. In accordance with the principal characteristics delineated in the preceding section, the pertinent motivation items have been devised, which primarily assess the capacity to discern and comprehend one’s own emotions and evaluate the ability to perceive the emotions of others, as shown in Table 1.

**Table 1:** Emotional intelligence scale.

Serial number	Measurement items
EI1	I will praise others when I find that they are doing well in some aspect of physical activity.
EI2	I want to be able to do most of the physical activity programs I want to do.
EI3	When doing a physical activity, I motivate myself by visualizing it being completed beautifully.
EI4	I will be in a good mood to face difficult physical activities.

EI, emotional intelligence.

#### 3.3.2 Achievement motivation scale

In this study, we utilize the Achievement Motivation Scale, as compiled by Hermans (32, 33), to assess the achievement motivation of university students. This scale is divided into two factors: convergent and avoidant. It measures the tendency of university students to pursue high standards of athletic achievement, in terms of the motivation to hope for success and the motivation to avoid failure, respectively. These findings are shown in Table 2.

**Table 2:** Achievement motivation scale.

Serial number	Measurement items
AM1	I worry about failing when it comes to completing what I consider to be difficult physical activities.
AM2	I do not like physical activities that measure my abilities.
AM3	I do not like doing physical activities that I do not know if I can do, even if no one else does.
AM4	Those physical activity programs that seem quite difficult worry me when I do them.
AM5	I am apprehensive about physical activities that I am not sure I can handle.

AM, achievement motivation.

#### 3.3.3 Interpersonal relationships scale

In this study, we make use of the Interpersonal Relationships Scale, developed by Garthoeffner (34), as a means of assessing the interpersonal relationships of university students. The scale was developed on the basis of Buhimester (35) and is widely used to measure the current status of university students’ interpersonal relationships, as shown in Table 3.

**Table 3:** Interpersonal relationships scale.

Serial number	Measurement items
IR1	Participating in physical activities with a large group of teammates, often feeling isolated or lost.
IR2	There is no one to talk to about the problems you face when you are involved in physical activities.
IR3	I was overly envious and jealous of other people’s athletic achievements.
IR4	I am unable to complete a physical activity with others in a shared friendly manner.
IR5	I have been rejected and ignored by others in physical activities.

IR, interpersonal relationships.

#### 3.3.4 Physical activity engagement scale

In this study, the Physical Activity Rating Scale (PARS), which was designed by Hashimoto (1990) (36) and translated and revised by Liang and Liu (1994) (37), was employed to assess the physical activity input of university students. The scale encompasses three key aspects: exercise intensity (EI), time (ET) and frequency (EF). The scoring method for physical activity is as follows: AE = EI × (ET - 1) × EF, with a score ranging from 0 to 100. The level of exercise was classified as follows: low (AE ≤ 19), moderate (20 ≤ AE ≤ 42) and high (AE ≥ 43).

### 3.4 Statistical analysis

IBM SPSS Statistics 26.0 was used for descriptive analysis of relevant variables, correlation analysis, and AMOS was also used to analyze the mediating effects of Achievement Motivation and Interpersonal Relationship between Emotional Intelligence on Physical Activity Engagement; Cronbach’s alpha coefficients and validation factor analyses were used for the reliability tests, respectively. In this study, a total scale containing all items was established, and in order to ensure that the internal consistency of the scale was guaranteed, the internal consistency reliability coefficients of the scale were tested for internal consistency with Cronbach’s *α* values before conducting the validation factor analysis. In the event that the correlation between the item and the dimension is low, and the overall reliability of the remaining items in the dimension is significantly enhanced following their removal, the item is deleted.

## 4 Results

### 4.1 Reliability testing

As shown in Table 4, both the Cronbach’s *α* and CR values exceeded 0.7, thereby indicating that each measurement model exhibited satisfactory internal consistency reliability. The factor loadings of all observed question items were greater than 0.7, and the AVE values of the latent constructs were greater than 0.5, indicating that the measurement models exhibited good convergent validity.

**Table 4:** Summary results for confirmatory factor analysis of all factors and items.

Variable	Item	Std	Cronbach’s *α*	CR	AVE
Emotional intelligence	EI1	0.911	0.952	0.873	0.565
	EI2	0.930			
	EI3	0.923			
	EI4	0.960			
Achievement motivation	AM1	0.953	0.950	0.856	0.521
	AM2	0.902			
	AM3	0.919			
	AM4	0.901			
	AM5	0.913			
Interpersonal relationships	IR1	0.923	0.972	0.732	0.509
	IR2	0.933			
	IR3	0.906			
	IR4	0.853			
	IR5	0.849			
Physical activity engagement	PAE1	0.750	0.811	0.824	0.556
	PAE2	0.909			

Std, Standardized factor loading coefficients.

### 4.2 Validity testing

As shown in Table 5, the KMO value is 0.893, exceeding the 0.5 threshold, and the chi-square of Bartlett’s test of sphericity is 9374.496, indicating that the indicators are interrelated and suitable for factor analysis. As shown in Table 6, the factor loadings of each item on its associated variables were greater than 0.5, while the factor loadings of the crossover variables did not exceed 0.5. This indicates that the questionnaire has good structural validity.

**Table 5:** KMO and Bartlett’s sphere test.

KMO and Bartlett’s sphere test		
Measure of sampling adequacy		0.893
Bartlett’s sphere test	chi-square test	9374.496
	df	120
	P	0.000

*x2, chi-square test; df, degrees of freedom.*

**Table 6:** Results of exploratory factor analysis.

Total variance explained									
Item	Initial eigenvalues			Extraction sums of squares			Rotational load sum of squares		
	Total	Variance%	Cumulative%	Total	Variance%	Cumulative%	Total	Variance%	Cumulative%
1	7.277	45.483	45.483	7.277	45.483	45.483	4.568	28.553	28.553
2	2.959	18.495	63.978	2.959	18.495	63.978	4.185	26.156	54.709
3	1.665	10.407	74.385	1.665	10.407	74.385	2.822	17.637	72.345
4	1.354	8.461	82.846	1.354	8.461	82.846	1.680	10.501	82.846

As shown in Table 6, the factor loadings of each item on its associated variables were greater than 0.5, while the factor loadings of the crossover variables did not exceed 0.5. This indicates that the questionnaire has good structural validity.

### 4.3 Structural model

**Figure 2:**
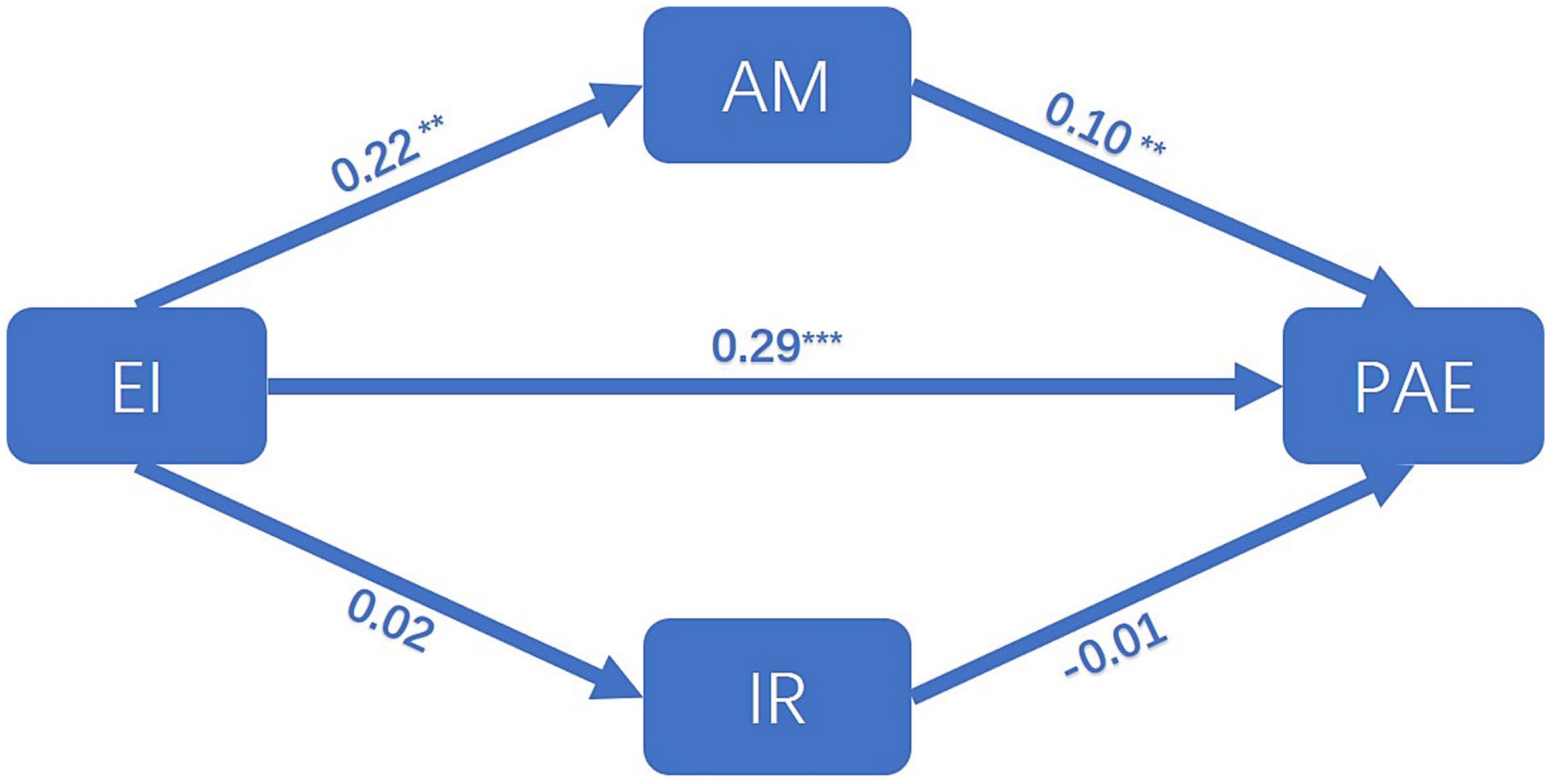
Structural model. (Chi-square = 613.694, df = 99, Chi-square/Df = 6.198, GFI = 0.893, CFI = 0.950, AGFI = 0.854). EI, emotional intelligence; AM, achievement motivation; IR, interpersonal relationships; PAE, physical activity engagement. **p < 0.005; ***p < 0.001.

In this study, Amos data processing software was employed to ascertain the statistical significance of the path coefficients. A structural model was constructed for the purpose of testing the hypotheses, as shown in Figure 2. The structural model demonstrated an acceptable fit to the data (Chi-square = 613.694, df = 99, Chi-square/Df = 6.198, GFI = 0.893, CFI = 0.950, AGFI = 0.854). The results of the study are presented in Figure 2, which demonstrates that there is a notable positive effect of EI on AM (*β* = 0.22, *p* < 0.05). The results indicate that EI has a significant positive effect on PAE (*β* = 0.29, *p* < 0.001). Furthermore, AM has a significant positive effect on PAE (*β* = 0.10, *p* < 0.05). However, the data suggest that EI has no effect on IR and that IR has no effect on PAE (*β* = 0.02, *p* = 0.741; *β* = −0.01, *p* = 0.851). Thus hypotheses 1, 2, and 3 were supported. Hypotheses 5, and 6 were not supported.

### 4.4 Testing for the mediation model

The mediation model was tested using bias-corrected nonparametric percentage Bootstrap 95% confidence intervals (CI) to ascertain the extent to which the hypothesized mediation role was borne out. As shown in Table 7, the mediating effects were found to be statistically significant for both the direct and indirect effects, and the ‘EI → AM → PAE’ pathway was fully mediated. Nevertheless, the mediating effect of IR between EI and PAE was not found to be significant. Thus hypothesis 4 was supported. Hypothesis 7 was not supported.

**Table 7:** Results of exploratory factor analysis.

Variable	ES	Product of coefficient		Bias-corrected 95% CI	
		SE	Z	Lower	Upper
IE	0.117	0.014	0.001	−0.503	0.004
EI → AM→PAE	0.195	0.03	0.001	0.141	0.261
EI → IR → PAE	0.116	0.027	0.760	−0.084	0.105
DE	0.192	0.052	0.001	0.192	0.395
TE	0.595	0.049	0.001	0.140	0.383

## 5 Discussion

The issue of undergraduate students engaging in physical activity is becoming a topic of growing interest for coaches, educators, and sports psychologists (38–40). Nevertheless, there is a paucity of research examining the relationship between emotional intelligence and the physical activity engagement of undergraduate students, as well as the potential mediating influences. Consequently, our study will facilitate a scientific and systematic comprehension of the evolving patterns of PAE by examining the factors that influence PAE and provide a foundation for addressing some of the issues surrounding physical inactivity among undergraduate students. The primary aim of this study is to investigate the effects of EI on university students’ PAE for the first time. The second aim was to elucidate the Mediating Role of AM and IR between EI and PAE. In light of the aforementioned considerations, this study undertook a questionnaire survey of university students in Shandong Province. The findings indicated that emotional intelligence exerted a direct influence on physical activity engagement, while only achievement motivation played a significant mediating role and interpersonal relationships did not.

### 5.1 Emotional intelligence and physical activity engagement

Emotional intelligence (EI) is an individual’s capacity to recognize, comprehend, regulate, and respond to their own and others’ emotions in an appropriate manner (41). The concept was initially proposed by psychologists Peter Salovey and John Mayer (42) and subsequently advanced and disseminated by Daniel Goleman (43). EI plays an important role in an individual’s social interactions and emotional life. It is closely related to a number of key areas, including interpersonal relationships, mental health, and professional success (44). The importance of EI cannot be overstated. It affects not only an individual’s emotional health and well-being but also has a significant impact on their performance at work and in life. Those with high EI are frequently better equipped to cope with stress, navigate complex social situations, and assume an active role in teamwork (45, 46).

The results of this study indicate that EI has a significant and positive effect on PAE. This may be due to the fact that individuals with high levels of EI are better able to manage and regulate their emotions, thereby making it easier for them to overcome difficulties and challenges encountered in physical activity (44, 47). This is also consistent with the findings of Mikolajczak et al. (48) and Petrides et al. (49), which indicate that individuals with high EI are generally more self-motivated. This allows them to maintain higher levels of motivation and persistence in the face of physical activity. Therefore, this study provides further evidence to support the importance of EI in promoting physical activity.

It is therefore recommended that coaches, parents, and other stakeholders focus their efforts on enhancing the EI of university students in order to effectively increase their level of PAE. This may be achieved by providing diversified, interesting, and challenging sports programs that stimulate individuals’ interest and enthusiasm, thereby complementing the relationship between EI and PAE (50–52). This study corroborates the positive correlation between EI and university students’ PAE, thereby underscoring the pivotal role of EI in fostering exercise behavior. These findings not only enhance the theoretical foundation of sport psychology but also provide crucial guidance for practice, enabling the design of more effective physical activity strategies.

### 5.2 The mediating role of achievement motivation and interpersonal relationships

Achievement motivation (AM) can be defined as an internal drive that is expressed by individuals in the pursuit of success, the attainment of goals, the overcoming of difficulties, and in competition with others (53). The Achievement Motivation Theory was initially proposed by psychologist David McClelland (54), who posited that AM represents a primary motivating factor influencing individuals’ work ethic and is closely associated with their success and achievement (55). The degree of AM has a significant impact on an individual’s performance in learning, work, and life.

The findings of our study indicate that university students’ AM acts as a mediator between EI and PAE. This finding is consistent with the results reported by Deci & Ryan (56) and Schutte et al. (31). It may be posited that individuals who demonstrate high levels of emotional intelligence typically possess a stronger drive for achievement and a tendency to set and pursue challenging goals (57). This intrinsic motivational drive prompts them to demonstrate elevated levels of engagement and persistence in physical activities (58). This finding is also consistent with the tenets of self-determination theory, which posits that an individual’s achievement motivation can exert a significant influence on their behavioral performance (56, 59). Therefore, the findings of this study serve to further validate the role of AM as a crucial link between EI and PAE.

However, this study did not find a significant mediating role in interpersonal relationships between emotional intelligence and physical activity. This finding may be related to a number of factors. Firstly, although emotional intelligence may help individuals establish and maintain good interpersonal relationships (57, 60), these relationships do not necessarily translate directly into physical activity engagement. Some research suggests that interpersonal relationships have a greater impact on mental health and well-being (61) than specific behavioral manifestations of physical activity. In addition, physical activity among university students may be more influenced by other factors such as time management, course load, and personal interests (62). Therefore, interpersonal relationships did not show a significant mediating effect in this process.

Therefore, it is suggested that coaches, parents, and other stakeholders can also use the mediating relationship of students’ achievement motivation between emotional intelligence and physical activity to effectively increase their participation in physical activity. Developing individuals’ emotional intelligence by setting small achievable goals and providing positive feedback and support can also significantly increase their participation (63, 64). It is also recommended that relevant sports psychology researchers and practitioners further investigate why interpersonal relationships do not mediate the effect and what influences it, in order to introduce new solutions. This study confirmed the mediating relationship of achievement motivation between emotional intelligence and physical activity participation among university students, thus highlighting the role of achievement motivation in promoting exercise behavior. These findings not only strengthen the theoretical foundation of sport psychology but also provide important guidance for practice and help to design more effective physical activity strategies.

### 5.3 Practical implications

This study highlights the significant impact of (EI) on PAE, particularly through the mediating role of AM. These findings have practical implications for the design of health interventions in universities. First, enhancing EI in undergraduate students may increase their PAE and potentially improve their overall health literacy and quality of life (65, 66). In addition, integrating EI with AM provides a new perspective for comprehensive interventions that combine mental health and physical education. By focusing on techniques such as emotional regulation and goal setting, students may experience greater self-efficacy and social support in the context of physical activity, which may contribute to more sustained engagement (67). Finally, this study provides practical insights for educational policy development, particularly in curriculum design within universities. Integrating modules that address both mental and physical health could promote the holistic development of students, potentially increasing engagement in physical activity and supporting mental well-being (68). The interplay between EI, AM thus not only broadens the theoretical understanding of physical activity, but also offers valuable directions for education and health policy in practice.

### 5.4 Research insights and limitations

In the context of sedentary and physically inactive university students, it would be useful to elucidate the mechanisms and subtle ways in which emotional intelligence influences the motivational and interpersonal aspects of university students’ sports participation in order to maintain and improve their physical and mental health, and to provide recommendations for fostering supportive sports environments and student well-being within the university community as a contribution to a healthy and happy life. The interaction and communication between the three elements of sport (family, athlete, and coach) is crucial for motivating young people to participate in physical activity and for the development of their physical and mental health.

In this paper, we investigated the mechanism of the influence of emotional intelligence on physical activity engagement mediated by achievement motivation and interpersonal relationships through a survey of emotional intelligence and physical activity engagement of university students in Shandong Province. There are some limitations of this study, firstly, although Shandong is one of the more populous provinces in China, the study of university students in Shandong cannot reflect all university students in China, which may affect the generalizability of the results, secondly, the sample of subjects in our investigation is small, future research can consider longitudinal studies with larger sample sizes to further validate the relationship between emotional intelligence and physical activity engagement. Finally, our study used a cross-sectional design, which limited our ability to establish causal relationships between variables. This is an inherent limitation when analyzing this type of data. Longitudinal or controlled studies are needed to reexamine the causal relationships along each pathway.

## 6 Conclusion

Our cross-sectional study represents a significant contribution to the existing body of research in the physical activity field. The study provides new evidence for physical activity engagement among university students, as well as indirect promotion through the mediating role of achievement motivation. It is imperative that educators, parents, and stakeholders are cognizant of the role of emotional intelligence in physical activity and utilize achievement motivation in an appropriate manner as a means of enhancing physical activity engagement among university students.

## Data Availability

The original contributions presented in the study are included in the article/supplementary material, further inquiries can be directed to the corresponding author.
